# Effect of the * Zataria multiflora* on Systemic Inflammation of Experimental Animals Model of COPD

**DOI:** 10.1155/2014/802189

**Published:** 2014-06-11

**Authors:** Mohammad Hossein Boskabady, Lilla Gholami Mhtaj

**Affiliations:** ^1^Neurogenic Inflammation Research Centre and Department of Physiology, School of Medicine, Mashhad University of Medical Sciences, Mashhad 9177948564, Iran; ^2^Pharmaceutical Research Center and Department of Physiology, School of Medicine, Mashhad University of Medical Sciences, Mashhad 9177948564, Iran

## Abstract

The effects of * Zataria multiflora *(*Z. multiflora*) on systemic inflammation in guinea pigs model of COPD were examined. Control animals, COPD (induced by exposing animals to cigarette smoke), COPD + drinking water containing three concentrations of the extract of *Z. multiflora,* and COPD + dexamethasone were studied (*n* = 6 for each group). Serum levels of IL-8 and malondialdehyde (MDA), total blood WBC (*P* < 0.01 for all cases), and eosinophil counts (*P* < 0.05) were higher and weight changes (*P* < 0.05) were lower in the COPD group compared to controls. IL-8 level (*P* < 0.001) and weight changes (*P* < 0.01 to *P* < 0.001) in all treated groups with *Z. multiflora* and total WBC number and MDA level in treated groups with two higher concentrations of the extract and lymphocytes percentage (*P* < 0.05) in the highest concentration of *Z. multiflora *and dexamethasone (*P* < 0.05 to *P* < 0.001) were significantly improved compared to the COPD group. Results showed a preventive effect of hydroethanolic extract from * Z. multiflora *on all measured parameters in animals model of COPD which was comparable or even higher (in the highest concentration) compared to the effect of dexamethasone at the concentration used.

## 1. Introduction

Chronic obstructive pulmonary disease (COPD) is an epidemic disease in the world [1] and smoking is an important risk factor for development of COPD [2]. COPD is characterized by two pathological features including bronchitis and emphysema. Inflammatory processes, oxidative damage, endothelial dysfunction, endothelial cell apoptosis, proteolysis, and vascular remodeling are lung tissue changes of COPD patients [2–4]. Some cells such as CD8+ cytotoxic, CD68+ macrophages, and neutrophils penetrate the airways and alveoli of COPD patients. These inflammatory cells release some proinflammatory mediators, including TNF*α*, interleukin-8, and macrophage inflammatory protein-1*α* (MIP-1*α*) which absorb more inflammatory cells and produce a positive inflammatory chain that preserves the inflammatory response [5, 6]. Another inflammatory response in COPD patients is an increase in number of CD68+ monocytes or macrophages in the bronchial mucosa [7, 8]. In severe COPD patients, an increase in the number of neutrophils is also observed [9, 10]. Therefore, antioxidants may have a role in the treatment of COPD [11].

*Z. multiflora* is a perennial plant with a woody, small leaves, fibrous root, and height of 40–80 cm, with highly narrow branches [12]. This plant has a limited distribution in the world and only grows in Iran, Pakistan, and Afghanistan [13].* Z. multiflora* contains various compounds of which some are bioactive chemicals, particularly terpenes such as thymol and carvacrol. Te plant also contains apigenin, luteolin, and 6-hydroxyluteolin glycosides, as well as di-, tri-, and tetramethoxylated. These compounds could be responsible for the therapeutic effects of* Z. multiflora* [12]. In Iran,* Z. multiflora* is used in traditional medicine for its antiseptic, analgesic, and carminative properties [14]. The effects of* Z. multiflora* essential oil on* Escherichia coli*,* Pseudomonas aeruginosa*, and* Staphylococcus aureus* have been described [15]. Antibacterial effects of this plant on the B. cereus [16], Staphylococcus aureus [17, 18], and Escherichia coli [18], antifungal [19], analgesic [20, 21], antioxidant [22], and immunoregulatory effects [23], its effect on gastrointestinal disorders [24, 25], and anti-inflammatory effects [26] have also been reported. The antispasmodic effect of the plant on different types of smooth muscle was also demonstrated [27–29]. Our previous study also showed relaxant effect of the other plant of this family (*Thymus vulgaris*) on tracheal smooth muscle [30]. The inhibitory effect of the extract of* Z. multiflora *and carvacrol on histamine (H1) [31] and muscarinic receptors [32–34] and its stimulatory effect on *β*-adrenoceptors [35] were demonstrated in our previous studies.

Therefore, in the present study, the effects of* Z. multiflora* on total and differential WBC in the blood, serum levels of IL-8 and MDA, and weight changes of guinea pig model of COPD have been examined.

## 2. Materials and Methods

### 2.1. Plant and Extract

*Z. multiflora* was collected from mountains in the fluorine mine in a region between Tabas and Yazd, Iran, and was identified by Mr. Joharchi and a sample was kept in the Herbarium of the Faculty of Sciences, Ferdowsi University of Mashhad (herbarium number: 35314). For the preparation of hydroethanolic extract from* Z. multiflora*, 125 g of dried shoots and powdered plant was mixed with 875 mL of 50% ethanol. The mixture was shaken for 72 hours at room temperature. The extract was then passed through the filter paper and the solvent was removed under reduced pressure. The dried extract was collected and kept at refrigerator temperature. Three concentrations (0.4, 0.8, and 1.6 mg/mL) of the extract were then used for treatment purpose. Extract concentrations were chosen according to the previous study [36–41].

### 2.2. Characterization of the Extract of* Zataria multiflora*

**Figure 1:**
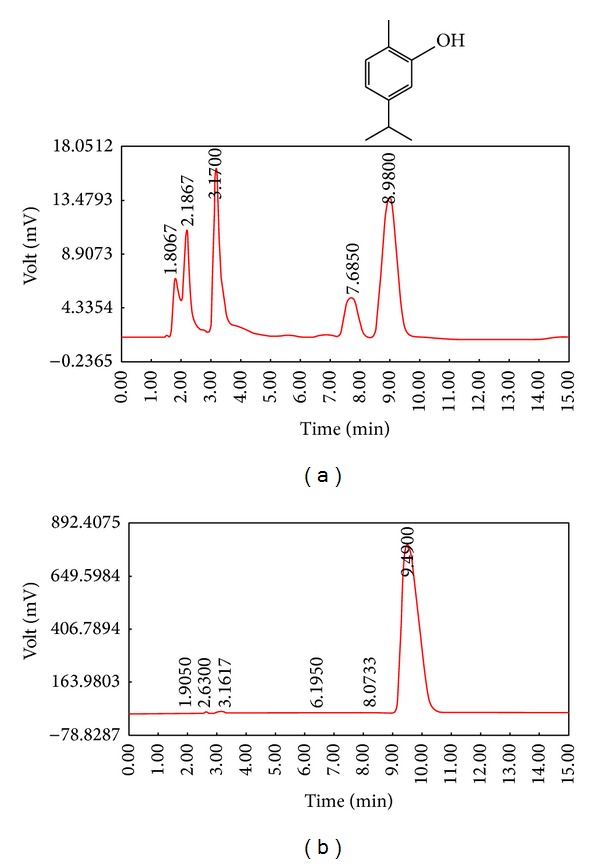
Fingerprint of (a) the extract of* Z. multiflora* (50 *μ*g/mL) and the (b) chromatographic profile of pure carvacrol (C_10_H_14_O, 5/1000) with retention time of about 9 min. MW = 150.217 [35].

In our previous study [35] the characteristic of the extract of this plant was identified, using HPLC (Waters 474, Waters Corporation, MA, USA) fingerprint. The extract was dissolved in the mobile phase and filtered through 0.22 *μ*m membrane filter. An aliquot (20 *μ*L) of sample (50 *μ*g/mL) was injected into the reverse phase HPLC column (C18; 250 × 4.6 mm). The mobile phase consisted of methanol : water (60 : 40) with an isocratic elution at the flow rate of 1 mL/minute. The peaks were monitored at 254 nm (Figure 1(a)).  Figure 1(b) illustrates the chromatographic profile of pure carvacrol (5/1000) with retention time of about 9 min. All used solvents were HPLC graded and supplied by Caledon Laboratories, Georgetown Ltd., Canada. Using the calibration curve, the quantification of carvacrol in a sample of the extract of* Z. multiflora* was achieved which was about 0.16% w/w (Figure 1).

### 2.3. Exposure of Animals to Cigarette Smoke

Exposure of guinea pigs to cigarette smoke was performed according to the method described previously [42, 43]. To create a model of chronic obstructive pulmonary disease (COPD) in guinea pigs, animals were placed in special boxes which included two parts: smaller part for the head to which cigarette smoke was administered (the animal's head) and larger part for the body of guinea pigs. Twenty-milliliter puffs of cigarette smoke were drawn out of the cigarettes using a syringe and then exhausted at a rate of two puffs per minute into the animals' head (every 30 seconds, one puff of cigarette smoke was transferred into the head part). Animals were exposed for 8-9 minutes to the smoke of one cigarette, and there was a 10-minute break between the two cigarettes. The animals were exposed initially to one cigarette per day and gradually increasing to a maximum of 5 cigarettes per day. The animals were exposed to cigarette smoke (Magna: Nicotine = 5, tar = 6) for 3 consecutive months, 5 days per week, and 5 cigarettes per day (the cigarettes' filters were not removed).

### 2.4. Animals and Groups

Thirty-six guinea pigs of both sexes (600–800 g) were used in this study. Animals were kept in a temperature-controlled room. The animals were divided into six groups in random order as follows (for each group, *n* = 6).

1. Control group: the animals were exposed to ambient air and received drinking water alone.
2. COPD group: the animals were exposed to cigarette smoke for 3 months and received drinking water alone.
3. COPD + dexamethasone: the animals were exposed to cigarette smoke and received drinking water containing dexamethasone (50 *μ*g/mL).
4. COPD +* Z. multiflora* dose 1: the animals were exposed to cigarette smoke and received drinking water containing the extract of* Z. multiflora* (0.4 mg/mL).
5. COPD +* Z. multiflora* dose 2: the animals were exposed to cigarette smoke and received drinking water containing the extract of* Z. multiflora* (0.8 mg/mL).
6. COPD +* Z. multiflora* dose 3: the animals were exposed to cigarette smoke and received drinking water containing the extract of* Z. multiflora* (1.6 mg/mL).

The extract or dexamethasone was added to the drinking water daily which was freely available for animals. The volume of uptake of drinking water was checked which was about 100 mL/day for each guinea pig in all groups.

### 2.5. Biochemical Parameters

#### 2.5.1. Measurement of Malondialdehyde (MDA)

After exposing the animal's chest, a 4 mL blood sample was taken from the heart and collected in a citrate tube. Then blood was centrifuged and the serum was separated and stored in −70°C until the end of the study to measure the concentrations of IL-8 and MDA in all groups. MDA level, as an index of lipid peroxidation, was measured. MDA reacts with thiobarbituric acid (TBA) as a thiobarbituric acid reactive substance (TBARS) to produce a red colored complex which has a peak absorbance at 535 nm. Two mL from reagent of TBA/trichloroacetic acid (TCA)/hydrochloric acid (HCL) was added to 1 mL of serum, and the solution was heated in a water bath for 40 minutes. After cooling, the whole solutions were centrifuged at 1000 g for 10 minutes. The absorbance was measured at 535 nm [44]. The MDA concentration calculations were performed using the following formula: *C*(*m*) = Absorbance/(1.56 × 105).

#### 2.5.2. Measurement of IL-8

To measure the concentration of IL-8, a double antibody sandwich enzyme-linked immunosorbent assay (ELISA) kit was used (Hangzhou Eastbiopharm Co., Ltd., Hangzhou) according to the manufacturer's protocol.

### 2.6. Total WBC and Differential WBC Measurement

After opening the animal's chest, a 4 mL blood sample was taken from the heart and was collected in the test tube containing anticoagulant EDTA. Total WBC was counted in duplicate in a hemocytometer (in a Burker chamber) in blood stained with a Turk solution (1 : 10 dilution consisted of 1 mL of glacial acetic acid, 1 mL of gentiac vialet solution 1%, and 100 mL distilled). Differential cell counts were done on thin slide, prepared with smearing the blood sample, using Wright-Giemsa stain. According to staining and morphological criteria, differential cell analysis was carried out under a light microscope by counting 100 cells, and the percentage of each cell type was calculated.

### 2.7. Animals Weight Measurement

Animals were weighted at the beginning of the study (before the first smoke inhalation) and after 3 months (at the end of exposure period); then the difference between the two weights was calculated.

### 2.8. Statistical Analysis

All data were expressed as mean ± SEM. Comparison between the results of COPD and control groups was performed using unpaired *t*-test. The data of treated groups were also compared with COPD group using unpaired *t*-test. The comparison between the data of animals treated with three concentrations of* Z. multiflora* was performed using ANOVA with Tukey-Cramer posttest. Significance was accepted at *P* < 0.05. All statistical analyses were made using GraphPad Instat version 3.00 (GraphPad Software, San Diego, CA, USA).

## 3. Results

### 3.1. Serum MDA Level

**Figure 2:**
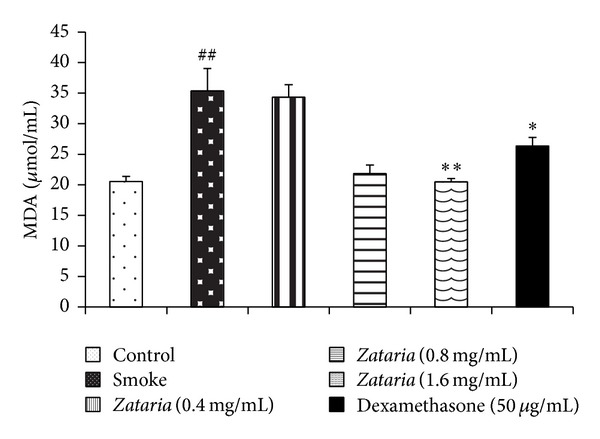
Serum level of MDA in control, COPD and COPD treated with dexamethasone, and three concentrations of* Z. multiflora*. Statistical differences between control versus COPD group: ^##^ *P* < 0.01. Statistical differences between COPD and treated groups: *P < 0.05, **P < 0.01.

Serum level of MDA in COPD group was significantly higher than in control group (*P* < 0.01). However, there was a significant reduction in serum levels of MDA in COPD groups treated by dexamethasone (*P* < 0.05) and two higher concentrations of* Z. multiflora* (*P* < 0.01 for both cases) compared to COPD group (Figure 2).

### 3.2. Serum Levels of Interleukin-8

**Figure 3:**
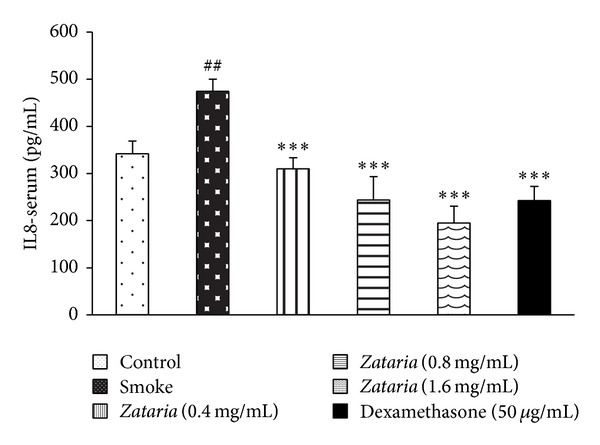
Serum level of IL-8 in control, COPD and COPD treated with dexamethasone, and three concentrations of* Z. multiflora*. Statistical differences between control versus COPD group: ^##^ *P* < 0.01. Statistical differences between COPD and treated groups: ***P < 0.001.

Serum levels of IL-8 were significantly increased in COPD group compared to control group (*P* < 0.01). However, in treated groups with dexamethasone and three concentrations of* Z. multiflora*, the level of IL-8 was significantly lower than in COPD group (*P* < 0.001, for all cases, Figure 3).

### 3.3. Total and Differential WBC Counts

**Figure 4:**
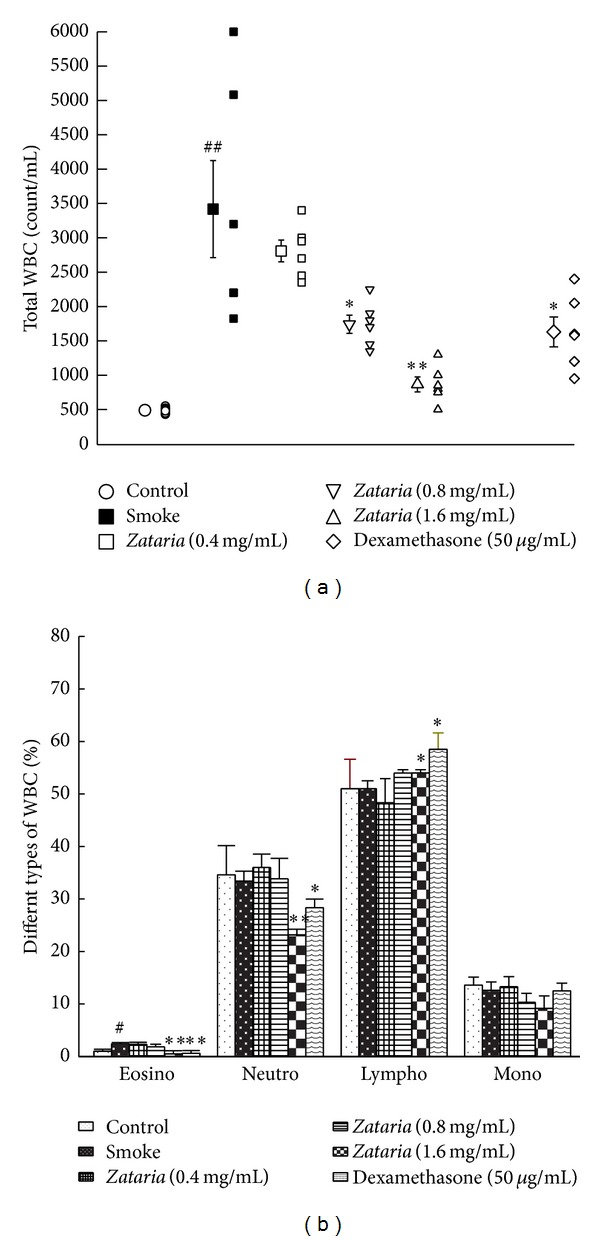
Total (a) and differential (b) WBC counts in control, COPD and COPD treated with dexamethasone, and three concentrations of* Z. multiflora*. Statistical differences between control versus COPD group: ^#^ *P* < 0.05, ^##^ *P* < 0.01. Statistical differences between COPD and treated groups: *P < 0.05, **P < 0.01.

Total WBC (*P* < 0.01) and eosinophil counts (*P* < 0.05) were significantly higher in COPD compared to control group (Figure 1). Total WBC number in treated groups with dexamethasone and two higher concentrations of* Z. multiflora*, eosinophil, and neutrophil percentage in the treated groups with dexamethasone and the highest concentration of the extract were significantly improved compared to COPD group (*P* < 0.05 to *P* < 0.01). In addition, lymphocyte percentage in treated groups with dexamethasone and the highest concentration of* Z. multiflora* was significantly increased compared to COPD group (*P* < 0.05, Figure 4).

### 3.4. Weight Changes

**Figure 5:**
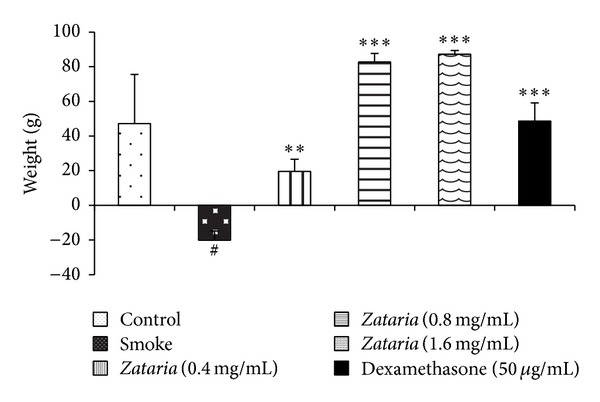
Weight changes in control, COPD and COPD treated with dexamethasone, and three concentrations of* Z. multiflora*. Statistical differences between control versus COPD group: ^#^ *P* < 0.05. Statistical differences between COPD and treated groups **P < 0.01, ***P < 0.001.

Weight changes at the end of three months of experimental period, in the COPD group, were significantly (*P* < 0.05) lower than in control group. However, weight changes in the treated groups with dexamethasone and all concentrations of* Z. multiflora* were significantly increased compared to COPD group (*P* < 0.01 to *P* < 0.001, Figure 5).

### 3.5. Comparison of the Effect of Three Concentrations of* Z. multiflora* with Dexamethasone

Total WBC number in treated group with the highest concentration of* Z. multiflora* was significantly lower, and weight changes in the two higher concentrations of the plant were significantly higher (*P* < 0.05 to 0.01) than in the dexamethasone group. In treated group with low concentration of the extract, total WBC, eosinophils, and neutrophils percentage as well as serum levels of MDA were higher but lymphocyte percentage and weight change were lower than in the treated group with dexamethasone (*P* < 0.05 to 0.01; Tables 1 and 2).

### 3.6. Difference between Three Concentrations of* Z. multiflora*

The effects of the two higher concentrations of* Z. multiflora* on MDA, total WBC, and weight change were higher than the effect of low concentration and the effects of the highest concentrations of the extract on eosinophil, neutrophil, and lymphocyte were significantly higher than the effect of its low and medium concentration (*P* < 0.05 to 0.01; Tables 1 and 2).

## 4. Discussion

In the present study, the preventive effect of* Z. multiflora*, in a guinea pigs model of COPD by their exposure to cigarette smoke, was studied. Increased interalveolar septum, increased lymphatic tissue in the lung parenchyma, the destruction of alveolar wall, and existence of emphysema in the lung and intra-alveolar bleeding in almost all animals exposed to cigarette smoke were observed, using similar method of exposure to cigarette smoke, which clearly suggest an animal model of COPD [42, 43]. According to previous studies, IL-8 was also used as an inflammation factor in COPD patients [45–48]. Total and differential WBC counts as well as serum level of IL-8 were measured in control, nontreated, and treated COPD groups to evaluate systemic inflammation. Serum level of MDA was also measured to evaluate oxidant condition. The weight change of animals of different groups during the study period was also examined. The results showed increased total WBC and eosinophils count in COPD compared to control group. Serum levels of IL-8 were also increased in COPD compared to control group. The mortality rate in the present study was zero and all animals in all groups survived until the end of the study.

Increased total and eosinophils counts as well as serum level of IL-8 indicated a systemic inflammation in animals exposed to cigarette smoke (an animal model of COPD). Systemic inflammation is a well-known phenomenon in COPD patients [46] and animal model of COPD [47]. Previous studies have also shown that inflammatory cells including macrophages, neutrophils, and T lymphocytes play a key role in COPD. Studies have also shown that the eosinophils, as a source of cytokines IL-3, -4, -5, -6, and -8, eosinophil-derived neurotoxin, eosinophil peroxidase, matrix metalloproteinase, and reactive oxygen species have an important role in the pathogenesis of COPD [48]. An increase in serum IL-8 level in smokers with COPD compared to healthy people was also observed previously [49]. The results of the present study also showed increased serum level of MDA which indicated oxidative stress in COPD group. In a previous study, the MDA level was also higher in COPD patients than in normal subjects which supports the results of the present study [50, 51]. The results showed that weight gain in the exposed animals to cigarette smoke during three months was significantly lower compared to control group. Mozzaffarian also showed the effect of cigarette smoking on body weight loss similar to findings of the present study [52]. Increased total and differential WBC counts, IL-8, MDA, and weight change in exposed animals to cigarette smoke showed the induction of an animal model of COPD in the present study and indicated a systemic inflammation in animal model of COPD.

According to traditional medicine,* Z. multiflora* has a beneficial effect on the cough due to colds and bronchial inflammation [53]. Therefore, in the present study, the therapeutic effects of* Z. multiflora* in an animal's model of COPD were evaluated. Treated COPD animals with all the three concentrations of the extract prevented increased serum level of IL-8 and weight change but total WBC and neutrophils counts as well as MDA level decrease in COPD animals treated with the two higher concentrations of the extract. In addition, eosinophils count was also decreased in only treated animals with the highest concentration of the extract. Therefore, the results suggest that extract of* Z. multiflora* has a preventive effect on systemic inflammation in animal model of COPD. In this study, serum levels of MDA were also decreased by the extract of* Z. multiflora*. The effect of the extract on MDA seen in the present study confirms the results of previous studies which have also demonstrated antioxidant effect of* Z. multiflora* [22].

The effect of* Z. multiflora* on all measured parameters was concentration dependent and the effects were increased with increasing extract concentration. The effects of medium and especially high concentrations of the extract were greater than the effect of its low concentration. In addition, the effects of high concentration of the extract on some parameters were also greater than its medium concentration. Concentration-dependent effects of the extract also confirm anti-inflammatory effects of* Z. multiflora*. The effects of the three concentrations of the extract were studied according to the previous studies [36–41] and, using these concentrations, a plateau in the effect of the extract was not achieved.

Comparable effects of the extract with those of dexamethasone are other important reasons for the anti-inflammatory mechanism of* Z. multiflora*. Although the effects of low concentration of the extract were less than dexamethasone, the effects of its high concentration on almost all parameters and the effects of the medium concentration on some parameters were significantly greater than the effects of dexamethasone at used concentrations. In the present study, dexamethasone was used as positive control treatment in COPD according to several previous studies [54–56].

In fact, the previous studies have shown the anti-inflammatory effects of* Z. multiflora*, which support the results of the present study [26]. Antitussive effect [53] of plant also could be due to its anti-inflammatory mechanism and confirm the results of the present study. Our previous studies also showed the effect of the extract on Th1/Th2 balance (IFN-*γ*/IL4 ratio) toward increasing Th1 subtype activity on both sensitized animals and human mononuclear cells [36] as well as its effect on total and differential WBC count and endothelin level in blood of ovalbumin sensitized guinea pigs [37] which may confirm the immunoregulatory and anti-inflammatory effects of the plant. The effect of carvacrol, the main constituent of the plant, on tracheal responsiveness, inflammatory mediators, total and differential WBC count in blood [38], and serum cytokines and endothelin levels [39] in sensitized guinea pigs was also shown which can support the anti-inflammatory effect of* Z. multiflora.* Therefore, these results suggested an anti-inflammatory and antioxidant activity which lead to a preventive effect for* Z. multiflora* on systemic inflammation in an animal model of COPD (exposed animals to cigarette smoke).

In addition, the protective effect of the plant on inflammatory bowel disease [25], the effect of aqueous and ethanolic extracts from the aerial parts of* Z. multiflora* on acute and chronic inflammation [26], and preventive effect of the plant, including total extract, flavonoid fraction, and the essential oil, on carrageenan (CAR) induced rat paw edema [55], have been demonstrated. Antioxidant [22] and immunoregulatory [23] effects of the plant have also been shown. All these studies indicate anti-inflammatory and antioxidant activity of the plant and support the findings of the present study. However, the effect of different constituents of the plant on animal model of COPD as well as the effect of the extract and its constituents on COPD patients should be examined in further studies.

As it is obvious, all aspects of inflammation could not be examined in a single study. Therefore, the effect of the extract on other cytokines, inflammatory mediators, and biomarkers such as ED-1 should be examined in further studies. In the present study, the effects of only three concentrations of the extract were examined which were chosen based on the previous studies [36–41] which were insufficient for determination of IC50 for extracts and its effective dose. Therefore, IC50 for extracts and its effective dose should be examined in further studies.

The preferred route of administration of drugs for treatment of respiratory disorders is inhalation. However, inhaler drugs should be disinfected. In addition, their particle size and flow rate should be known and their proper use should be standardized to ensure the penetration of drugs to the lung. Achievement of these characteristics for a plant extract is very difficult specially the sterility, particle size, and proper use in animals. Therefore, in the present study, the extract was dissolved in drinking water of animals and administered orally similar to our previous studies [36–39, 57, 58]. However, in further studies, the effect of standardized inhaled extract should be examined. In addition, we are going to examine the effect of the extract of this plant on asthmatic and COPD patients which will try to use inhaled extract.

Due to the existence of various constituents, it is very difficult to study the pharmacokinetic of the extract globally. However, the pharmacokinetic of orally administered thymol and carvacrol showed half-lives in total digestive tract that ranged between 1.84 and 2.05 h. Both of them were almost completely absorbed in the stomach and the proximal small intestine. Plasma concentrations (sum of free and conjugated compounds) peaked at 1.39 and 1.35 for carvacrol and thymol which was accompanied by high concentrations in urine. In addition, carvacrol and thymol were not degraded in jejunal simulations, but about 30% losses were found in caecal simulations [59]. However, the pharmacokinetic of other constituents of the plant should be studied in further studies.

## 5. Conclusion

In conclusion, the results of this study indicated a preventive effect of* Z. multiflora* on total and differential WBC, serum levels of MDA and IL-8, and weight change in an animal model of COPD which was comparable or even more potent than the effect of dexamethasone at used concentrations. Therefore, the results suggest a preventive therapeutic effect for* Z. multiflora* on systemic inflammation in COPD.

## Figures and Tables

**Table 1:** Weight changes (g) and serum levels of MDA (µg/mL) and IL-8 (pg/mL) in an animal model of COPD treated with three concentrations of *Z. multiflora* (0.4, 0.8, and 1.6 mg/mL) and dexamethasone (50 µg/mL).

Parameter	*Zataria* (0.4 mg/mL)	*Zataria* (0.8 mg/mL)	*Zataria* (1.6 mg/mL)	Dexamethasone
Weight changes	19.5 ± 7.13^+^	82.66 ± 4.97^+,###^	87.16 ± 2.21^++,###^	47.16 ± 9.43
MDA	34.337 ± 2.035^++^	21.848 ± 1.395^###^	20.491 ± 0.535^+,###^	26.276 ± 1.362
IL-8	309.93 ± 23.22	243.91 ± 49.19	194.91 ± 35.91	241.51 ± 30.19

Values are presented as mean ± SEM. Statistical comparisons between COPD groups treated with *Z. multiflora* and dexamethasone: ^+^ *P* < 0.05, ^++^ *P* < 0.01. Statistical comparison was done using unpaired *t*-test. Statistical comparison between the effect of two higher concentrations of the extract (0.8 and 1.6 mg/mL) with its low concentration (0.4 mg/mL): ^###^ *P* < 0.001. The statistical comparison was done using ANOVA with Tukey-Kramer multiple posttest.

**Table 2:** Total number and differential percentage of WBC count in an animal model of COPD treated by three concentrations of *Z. multiflora* (0.4, 0.8, and 1.6 mg/mL) and dexamethasone (50 µg/mL).

Parameter	*Zataria* (0.4 mg/mL)	*Zataria* (0.8 mg/mL)	*Zataria* (1.6 mg/mL)	Dexamethasone
Total WBC	158.86 ± 2808.33^+++^	90.75 ± 1741.66^###^	62.8 ± 866.66^++,###,¶¶^	216.86 ± 1630
Monocyte	13.33 ± 1.05	10.33 ± 1.83	9.1 ± 0.87	12.5 ± 1.86
Neutrophil	36 ± 1.77^+^	33.83 ± 1.28	33.16 ± 1.13^##,¶¶¶^	28.66 ± 1.22
Lymphocyte	48.33 ± 2.18^++^	54 ± 1.39	67.16 ± 1.94^#,++^	58.5 ± 1.77
Eosinophil	2.33 ± 0.33^++^	1.83 ± 0.4^#^	0.5 ± 0.22^###,¶^	0.66 ± 0.33

Values are presented as mean ± SEM. The data of total WBC is their count in one mL of blood and those of each type are the percentage of total WBC. Statistical comparisons between COPD groups treated with *Z. multiflora* and dexamethasone: ^+^ *P* < 0.05, ^++^ *P* < 0.01, ^+++^ *P* < 0.001. Statistical comparison was done using unpaired *t*-test. Statistical comparison between the effect of two higher concentrations of the extract (0.8 and 1.6 mg/mL) with its low concentration (0.4 mg/mL): ^#^ *P* < 0.05, ^##^ *P* < 0.01, ^###^ *P* < 0.001. Statistical comparison between the effect of high concentration of the extract (1.6 mg/mL) with its medium concentration (0.8 mg/mL): ^¶^ *P* < 0.05, ^¶¶^ *P* < 0.01, ^¶¶¶^ *P* < 0.001. The statistical comparison was done using ANOVA with Tukey-Kramer multiple posttest.
